# The use of animal by-products in a circular bioeconomy: Time for a TSE road map 3?

**DOI:** 10.1016/j.heliyon.2023.e14021

**Published:** 2023-03-06

**Authors:** Nathan Meijer, Leo W.D. Van Raamsdonk, Elise W.J. Gerrits, Marko J. Appel

**Affiliations:** Wageningen Food Safety Research (WFSR), Akkermaalsbos 2, 6708 WB Wageningen, the Netherlands

**Keywords:** Sustainability, Animal proteins, Legislation, TSE, EU, European Union, TSE, Transmissible Spongiform Encephalopathy, BSE, Bovine Spongiform Encephalopathy, CJD, Creutzfeldt-Jakob disease, EFSA, European Food Safety Authority, WOAH, World Organization for Animal Health, EURL, European Reference Laboratory for Animal Proteins, PAP, Processed Animal Protein, PCR, Polymerase Chain Reaction, QRA, Quantitative Risk Assessment

## Abstract

In 2005 and 2010, the European Commission (EC) published two subsequent ‘Road Maps’ to provide options for relaxation of the bans on the application of animal proteins in feed. Since then, the food production system has changed considerably and demands for more sustainability and circularity are growing louder. Many relaxations envisioned in the second Road Map have by now been implemented, such as the use of processed animal proteins (PAPs) from poultry in pig feed and vice versa. However, some legislative changes, in particular concerning insects, had not been foreseen. In this article, we present a new vision on legislation for increased and improved use of animal by-products. Six current legislative principles are discussed for the bans on animal by-products as feed ingredients: feed bans; categorization of farmed animals; prohibition unless explicitly approved; approved processing techniques, the categorization of animal by-products, and monitoring methods. We provide a proposal for new guiding principles and future directions, and several concrete options for further relaxations. We argue that biological nature of farmed animals in terms of dietary preferences should be better recognised, that legal zero-tolerance limits should be expanded if safe, and that legislation should be revised and simplified.

## 1 Introduction

Legislation in the European Union (EU) on the restrictions on the use of animal by-products was codified in 2001 by adopting Regulation (EC) No 999/2001. The primary reason for these restrictions was to contain the spread of Transmissible Spongiform Encephalopathies (TSEs), mainly Bovine Spongiform Encephalopathy (BSE, mad cow disease [1]; which had been associated with the presence of infected material in animal feed resulting from the recycling of proteins of animal origin in feed [2,3]. Human transmission of Creutzfeldt-Jakob disease (CJD) has been linked to consumption of BSE-infected meat [4,5]. In addition, the EU had witnessed epidemics of foot and mouth disease, as well as swine fever, that were related to the presence of animal by-products in feed [6,7]. In order to gain control over the BSE epidemic, several groups of measures were installed in Regulation (EC) No 999/2001, Regulation (EC) No 1069/2009, and subsequent implementing legislation (Regulation (EU) No 142/2011). These measures included prohibitions for using many types of animal by-products as feed material; compulsory treatment processes; monitoring prion presence in slaughtered animals; and requirements for labelling, storage, transport and trade.

Since 2001, a large epidemiological surveillance system to monitor for incidence of TSEs has been set up in the EU. Initially, almost all slaughtered bovine animals for human consumption were tested, but most Member States have now been allowed to implement revised monitoring programmes which only require them to test for the presence of TSEs in specific target groups of animals such as emergency slaughtered animals and fallen stock (Commission Decision 2009/719/EC). In 2017, the European Food Safety Authority (EFSA) concluded that there had been a significant decrease in classical BSE and that incidence in the EU could be considered low [8]. Since then, the situation in the EU has largely remained the same – although incidental single cases of classical BSE remain a cause for concern [9]. At a global level, most World Organization for Animal Health (WOAH) member states have a negligible risk status – except for Chinese Taipei, Ecuador, Greece, Russia, and the United Kingdom (except Northern Ireland) [10].

Prohibitions on the use of animal by-products as feed consist of three primary types of restrictions: a) the ruminant ban; b) the extended feed ban, and; c) the species-to-species ban. Certain animal by-products had been exempted from these bans from the start, such as dairy products and eggs - among others (Regulation (EC) No 999/2001, Annex IV, point 2). In addition, a series of later relaxations have been installed over the course of the years, such as the use of fishmeal in calf milk replacers (Regulation (EU) No 56/2013). Included in the Supplementary Materials in Ref. [11] is a complete timeline with major relaxations until 2019. Most recently, the use of processed animal proteins (PAPs) of poultry origin in pork feed and pig PAPs in poultry feed was approved, as was the use of ruminant collagen and gelatine in feed for non-ruminant farmed animals (Regulation (EU) 2021/1372 amending Regulation (EC) No 999/2001). In addition, the use of reared insects in feed for aquaculture animals (legal since 2017: Regulation No 2017/893) was extended to pig and poultry in Regulation (EU) 2021/1372. At this time, eight insect species (black soldier fly (*Hermetia illucens*), common housefly (*Musca domestica*), yellow mealworm (*Tenebrio molitor*), lesser mealworm (*Alphitobius diaperinus*), house cricket (*Acheta domesticus*), banded cricket (*Gryllodes sigillatus*), field cricket (*Gryllus assimilis*), and silkworm (*Bombyx mori*)) are permitted to be used in feed for these animals, but the substrate on which the insects may be reared is restricted to vegetable materials (i.e., not meat or fish).

The European Commission launched a first TSE Road Map in 2005 in order to provide options for relaxation of the installed restrictions for use as a feed ingredient, initially providing a short-term (2005–2009) and long-term (2009–2014) vision [12]. The basis to this process of gradual lifting the restrictions on the use of animal by-products were, and still are, four-fold: on-going publication of risk assessments by EFSA, balanced proposals for relaxations in concordance with the general principles, development of monitoring methods and societal requirements, and finally commitment of member states. Road Map 2 was published in 2010 [13], presenting a vision for the period 2010–2015. Both Road Maps showed a graphical overview of restrictions and legal applications in terms of source animal/material and target animal for consumption. A new Road Map 3 was not published in or after the year 2015. However, several major developments have taken place since 2010. Despite a similar risk status in terms of BSE incidence to other countries, as discussed above, the EU has lagged behind in implementing more permissive rules on the use of animal proteins in feed [14,15]. The rapidly increasing demand of sustainability and circular bioeconomy (the Green Deal, Farm to Fork) is a major shift in the mandate and priorities of legislators. We argue here that animal by-products should be reused to a larger extent. In the context of full circularity, every type of by-product should find a destination for reuse with a better ecological footprint in terms of nutrient recycling and production of greenhouse gasses [16]. In the view of the developments of the last five years, we consider a new TSE Road Map 3 a necessary guidance for future policies. This paper will propose this vision by evaluating the state-of-the-art and discussing legislative principles (sections 2, 3) and presenting directions for further relaxations. These future directions are firstly discussed in a general sense (section 4), and subsequently by providing specific options for relaxations (section 5).

## 2 Legislative principles and state-of-the-art

The current legislation on the use of animal proteins in feed is based on six different principles and elements. The first five principles are: 1) the feed bans; 2) the categorization of farmed animals; 3) prohibition unless explicitly approved; 4) approved processing techniques, and 5) the categorization of animal by-products. These first five aspects will be presented in this section; additionally, as sixth element, monitoring methods are discussed separately in section 3. These principles are used for an evaluation of future approaches and relaxations in the subsequent sections.

Firstly, the foundations of the legislative framework comprise three different bans: a) the permanent ruminant ban prohibiting the use of animal proteins in ruminant feed (Regulation (EC) 999/2001, Article 7, item 1), b) the permanent species-to-species ban prohibiting the use of animal proteins of a given source animal in feed intended for the same species (Regulation (EC) 1069/2009, Article 11, item 1a), and c) the extended feed ban prohibiting the use of animal proteins in a large range of other applications (Regulation (EC) 999/2001, Article 7, item 2). Exemptions have been installed for each of these three bans, but most of the relaxations as presented in the Road Maps 1 and 2 – in particular those put in force more recently – concern the extended feed ban (Regulation (EC) 999/2001, Annex IV). Important exemptions to the ruminant ban are the feeding of fish proteins to young ruminants (Regulation (EC) 999/2001, Article 7, item 3). One exemption to the species-to-species ban is the relaxation of caught fish, being a mixture of species, which is allowed to be fed to farmed fish of a species which might be included in the mixture of caught fish species (Regulation (EC) 142/2011, Annex VIII, Chapter II, item 2).

New derogations were put into force in 2021 (Regulation (EU) 2021/1372 amending Regulation (EC) 999/2001). The feeding of pig-PAP to poultry and vice versa had been under serious discussion since the 2010 TSE Road Map 2 [13]. However, at that time, monitoring methods for material of a certain (group of) species were not available. In 2019, the European Reference Laboratory for Animal Proteins (EURL-AP) finished the validation of a method for pig and one for chicken-turkey (excluding ducks and geese, although part of the definition of poultry; see Ref. [11]. A method for poultry, covering all species(-groups) of the definition of poultry, has been developed and tested successfully [17,18]. Validation of these methods allowed for differentiation between different PAPs and cleared the way for the authorisation of pig PAPs in poultry feed and vice versa (Regulation (EU) 2021/1372, preamble 12 and 13). The use of gelatine and collagen originating from ruminant material has been under discussion since 2005 [19]. After further evaluation by EFSA [49], these materials are now authorised as ingredients in feed for non-ruminants. For this evaluation, a probabilistic model was developed to estimate the BSE infectivity load, taking into account of different ‘risk pathways’ via which infected material could enter the food/feed chain. It was concluded that the probability that there would be no new cases of BSE was almost certain. Finally, based on the biological background of pigs and poultry, being omnivorous and (partly) insectivorous, respectively; insects have been authorised as ingredient in pig feeds and poultry feeds. The broader perspective of the biological background of the bans on animal by-products is discussed by Ref. [20].

Secondly, farmed animals are defined as one category and in a broad sense (Regulation (EC) No 1069/2009, Article 3, item 6). This definition includes animals for food and non-food production (fur animals); only pet animals are excluded. Clear differentiation is made between ruminant and non-ruminants in terms of permitted feed materials, but this broad definition necessitates highly comparable routes for gradual relaxations for most farmed animals despite biological differences in susceptibility and diet preferences.

Thirdly, the general principle of the legislation is to prohibit the use of animal proteins unless a specific relaxation is installed. This is an extension of the ‘precautionary principle’ (Regulation (EC) No 178/2002, Article 7). In the context of animal proteins, this is laid down in Regulation 999/2001, article 7. Relaxations following this general principle are provided in Annex IV of that Regulation. This Annex IV comprised less than one page in 2001, whereas the current version of Annex IV covers almost 30 pages and is highly complex. The consequence of this “prohibited, unless” principle is that new by-products start with case-by-case legalised application, which is a slow process. This is exemplified by the insect situation, which were legalised for aqua-feed only in 2017 and for poultry and pigs in 2021 – but a large number of legal barriers for the insect utilisation persist.

Fourthly, certain category 3 materials may be processed and used for feeding farmed animals. At this time; the subcategories of Category 3 (n) [*hides, skins, hooves, etc. of dead animals that did not show signs of zoonotic disease*], (o) [*adipose tissue from animals that did not show signs of zoonotic disease; slaughtered in a slaughterhouse; and considered fit for human consumption*], and (p) [*catering waste*] are not allowed to be used as basis for the production of PAPs and HPs (Regulation (EC) No 1069/2009, Article 14(d)(i)). These materials, as well as subcategory (m) [*parts of Rodentia and Lagomorphia*] are also not allowed to be processed into gelatine, hydrolysed proteins, or dicalcium or tricalcium phosphate (Regulation (EU) 142/2001, Annex X, Chapter II). Seven standard processing methods are defined In Chapter III of Annex IV of Regulation (EC) No 142/2011. Animal proteins of mammalian origin may only be manufactured into processed animal proteins (PAPs) intended for feeding to farmed animals by way of method 1; pressure sterilisation (Regulation (EU) 142/2001, Annex X, Chapter II, section 1). The process of fermentation is only mentioned in the context of use as petfood. The nutritional value of different types of PAPs must be carefully considered and evaluated for the composition of compound feeds to meet all nutritional requirements of the target animal [21]. This is also the case for reared insects such as fly larvae: although generally considered to be omnivorous, optimal yields and fatty acid profiles depend on composition of the substrate [22,23].

Finally, animal by-products are classified in three categories depending on their origin in terms of animal tissues or organs, type of processing and the degree of risk involved (Regulation (EC) 1069/2009, Articles 8–10; see Fig. 1.1 in Ref. [24]. Materials in category 1 consist of, for instance, animals suspected of being infected by a TSE. Category 2 materials consist of, for example, manure and animal by-products derived from animals which have been submitted to illegal veterinary medicines. The disposal and use of animal by-products and derived products varies per category (Regulation (EC) No 1069/2009, Articles 12–16). Only category 3 materials may be used for feeding farmed animals other than fur animals (Regulation (EC) 1069/2009, preamble 45). This category includes a variety of types of materials which are allowed to enter the food production chain as basis for several derived products. Processed animal proteins (PAPs; Regulation (EC) 142, 2011, Annex I, item 5; [50]) may be produced from the subcategories (a) to (l) (Regulation (EC) 142/2009, Annex X, Section 1, part A). An important derived product consists of hydrolysed proteins (HPs; Regulation (EC) 142, 2011, Annex I, item 14). This product can be produced from subcategories (a) to (l) as well (Regulation (EC) 142/2009, Annex X, Section 5, part A). At the other hand, the subcategories (m) to (p) are listed as source for the production of dicalcium phosphate, tricalcium phosphate or collagen (Regulation (EC) 142/2009, Annex X, Sections 6, 7 and 8). Subcategory (p), catering waste although included in Category 3, is prohibited for any application in the food production chain (Regulation (EC) 1069/2009, Article 11, item 1b). There are some legal applications for hydrolysed proteins, blood products, and gelatine. A more in-depth discussion on the applicability of hydrolysis for more sustainable use of animal proteins is discussed in Ref. [16].

## 3 Monitoring methods

The 6th element of the current legislation on the use of animal proteins in feed is related to monitoring methods. Animal by-products can be produced from a variety of sources in terms of animal species and types of tissue and are processed in multiple ways. This diversity of materials is monitored by only a few legally permitted methods of which the range of applicability is maximised. In principle, a diverse set of methods are available for legal monitoring, including: microscopy, DNA-based methods such as polymerase chain reaction-tests (PCR), protein-/antibody-based methods such as ELISA, and spectral analysis [11]. However, currently only two types of methods are legally authorised: microscopic detection and PCR (Regulation (EC) 152/2009 Annex VI). Both PCR and microscopy have proven to be suitable to monitor PAPs, which is a major animal by-product. Limitations of these methods are the lack of species identification down to the legal species groups of ruminants, pig and poultry (microscopy) and the lack of discriminating between prohibited and authorised materials of the same species (PCR: ruminant PAP vs. milk, pig PAP vs. blood products, poultry PAP vs. egg material, etc.).

The legislation of the use of animal by-products implies a zero-tolerance policy [25]. Consequently, the level of detection of monitoring methods should be as low as reasonably achievable (ALARA principle). The first technical limit for a monitoring method was 0.1% for microscopic detection, the only method available at the time (Directive 98/88/EC). This minimal required level of detection was carried over to subsequent versions of the legalised method for microscopy (Directive (EC) 2003/126/EC) and to other methods (PCR; Regulation (EC) 152/2009). The documented level of detection is much lower for both methods (microscopy: 0.005%, [26,27]; PCR: 0.0125%, [28]. The EURL-AP published a report on a ‘technical zero’ concept in 2017, which would be an action limit rather than a zero-tolerance policy [29]. As a consequence of implementing this ‘technical zero’ concept for ruminants, the risk of propagation of TSEs might be increased. Therefore, the EURL-AP calculated the mass fraction equivalent of the data generated by PCR methods, which allowed EFSA to calculate the cattle oral infectious dose and associated theoretical increase in BSE numbers per year in case porcine PAP were to be authorised in poultry feed – and vice versa – which was later permitted via Regulation (EU) 2021/1372, discussed in section 2 above (EFSA, 2018).

Besides PAPs (Regulation (EC) 142/2011, Annex I, Point 5) a set of other types of material, such as blood products, fat derivatives, milk products, gelatine and hydrolysed proteins, dicalcium phosphate, tricalcium phosphate, collagen, egg products and former food stuffs containing animal proteins (sections 1-10, respectively), is excluded from the definition of PAPs. This is acknowledged and discussed in the same Annex of Regulation (EC) 142/2011. Each of these types of materials require dedicated monitoring methods targeting at the appropriate legal parameters, since bone or muscle fragments for microscopy or DNA for PCR might be absent. Immunoassays (ELISA) are discussed in EFSA (2011) as not meeting the requirement of an LOD below 2%. This type of monitoring method is based on antibodies which can detect tissue-specific proteins, among other substances. Two immunoassays targeted at ruminant troponin have been validated for the detection of PAP at a level of 0.5%, which is four times lower than indicated by EFSA. Milk was not detected in this validation study [30,31]. These methods are capable of both animal-specific and tissue-specific detection of materials as demanded by the current legislation.

## 4 General future directions

Based on the legal principles and in the framework of the two permanent bans, the ruminant ban and the species-to-species ban, several modifications of legal principles can be considered for facilitating sustainability and circularity of the feed industry. The recognition of the biological nature of farmed animals in terms of diet, exemplified by the recognition of pigs as omnivorous animals and poultry as insectivorous animals in Regulation (EU) 2021/1372 (Preamble 16), should be extended to all farmed animals. This is most notably important for insects. At this time, eight insect species from a diverse spectrum of taxonomic orders are permitted to be included in animal feed. The types of feed matrices permitted for this large group of animal species – with a wide spectrum of feeding habits – should be diversified accordingly. Examples are the group of termites (Isoptera) that could be reared on materials containing lignin such as wood, garden products [32], and fly larvae (e.g., Diptera) for conversion of manure or manure-like materials [[33], [34], [35]]. Ringed worms (Annelids) have been proposed as a good source of proteins [36]: these worms feed partly on soil, but this type of feed material appears to be prohibited. In wider terms of legislation, the broad definition of farmed animals could be diversified to different groups of animals, primarily classified according to their feeding behaviour and their susceptibility to TSEs. A diversified classification of farmed animals would ease the authorisation of certain feeding strategies for defined groups of animals, especially in cases of larger biological distances between the source species of a feed material and the target species (consumer) [20].

The principle of zero tolerance in feed materials is supported by the ALARA principle and by the legal requirement of a limit of detection of 0.1%. In its updated quantitative risk assessment (QRA) of the BSE risk posed by PAPs, EFSA calculated the risk of accidental incorporation of infected ruminant PAP in ruminant feed (EFSA, 2018). It was concluded that a higher contamination level than the detection limit of 0.1% - under certain circumstances up to 2% - could be deemed acceptable as this would not lead to significant increases in BSE cases. A similar model could be developed for the risk of a low level of porcine PAP in pig feed, or poultry PAP in poultry feed. The primary safety concern of intraspecies recycling in case of poultry and pigs was the transfer of zoonoses or animal-specific diseases, such as swine fever (Regulation (EC) No 1774/2002). Ethical considerations against cannibalism in this context are valid, but that discussion is of a different nature than one on safety concerns. The same is true for concerns in the context of religious law (e.g., halal): both in case of inter- and intra-species recycling [37,38]. It has been shown that appropriate treatments of PAPs can reduce the virulence of a range of zoonoses by magnitudes of 10 up to 90 [39]. This aspect is further discussed in van Raamsdonk et al. (in press). Concerning the use of monitoring methods in a framework of varying LODs or technical limits, higher than 0.1% when applicable, monitoring methods other than PCR and light microscopy can provide added value. An overall requirement for a future use of an increasing set of animal by-products for circular bioeconomy and sustainability should be the development, validation and legal authorisation of a set of dedicated monitoring methods that are capable of identifying and adequately quantifying feed materials of different origin.

One of the basic principles of the current legislation of the feed ban is a general prohibition, with relaxations where possible. The aforementioned proposals (diversification of the definition of farmed animals and a diversification of the limit of detection depending on the specific situation) could be used as factors in a principle of ‘provisional authorisation’, i.e., safety assured for specific applications – as is for instance found in legislation on limits for undesirable substances in feed (Directive 2002/32/EC). We emphasize that in all cases, regardless of any type of authorization, feed ingredients should comply with the applicable restrictions for chemical and microbiological safety, for processing and for purpose (Regulation (EU) 68/2013, Preambles 2 to 5).

Finally, the current categorization of animal by-products in three categories according to their TSE risk is complicated and problematic. The derived products PAPs, HPs, blood products, milk products and egg products are all legally based on different subsets of subcategories of Category 3 (Regulation (EU) 142/2001, Annex X, Chapter II). While catering waste (subcategory (p) with the exemption of Category 1 (f)), is included in Category 3 as well, it is fully prohibited for feeding purposes. A clear categorization of animal by-products based on their potential use could be installed instead; for instance, with Category I materials' only use being for incineration, landfill, and possibly fertilizers. The use of Category 2 materials could be defined as limited to non-food and technical purposes, while all Category 3 materials’ main use would be for feed purposes. In the view of sustainability and circular bioeconomy demands, subcategories could shift to higher categories for better valorisation when technological and containment measures for assuring safety are progressing.

## 5 Options for specific new modifications and relaxations

Considering the legal principles and general directions towards full sustainability and circularity, a range of concrete proposals can be made for further relaxations. Proposals below are based on three prerequisites: safety, opportunities for monitoring the origin of the animal by-products (in most cases the source of the material, or the process when relevant), and options for management (physical separation of streams, either as pure material or as ingredient in compound feed). In all cases the required safety – in terms of prions, zoonoses, other microbiological hazards, accumulation of chemical hazards – has to be proven to be at a sufficiently high safety level according to the total set of applicable legislation.

1. The framework for permitted and not-permitted use of animal by-products in feed, depending on the ‘source animal’ and ‘intended consuming animal’, is shown graphically in Fig. 1 (upper part). Lagomorphs (rabbits, etc.) and other ruminants besides cattle (horses, camels) are outside of the scope of this figure. The ruminant ban is directed to the consuming animal and is reflected in the leftmost column being largely red. The species-to-species ban is indicated in the diagonal in the upper part of the scheme. Many combinations of source animal/material and target animal that are permitted are represented by green cells. A decreasing number of prohibitions remains from the extended feed ban (orange). The first proposals for relaxations are framed in the context of this current matrix. The letters for each of the four proposals (a, b, c, d) in the text below correspond to the letters indicated in the figure.
  a. Pig and poultry PAPs for insects. Reg. (EU) 2021/1372 permitted the feeding of insect PAPs to pigs and poultry, and the feeding of pig PAPs to poultry and vice versa. However, the use of insects as an intermediary between pigs and poultry (e.g., pig PAP- > insects PAP - > poultry) is not yet allowed. Reared insects acting as an intermediary in this process may not necessarily result in efficiency gains, but that in itself should not present a barrier for change. If containment can be ensured, monitored, and enforced, this practice should be permitted. At this time, we are unaware of any published literature on the capacity of insects to transfer any material of animal origin on which the insects are reared to the next step in the chain. It can be hypothesized that certain types of processing could affect such transmission, in particular the process of starving the insects prior to harvest to enable the insects to empty their gut contents. If transfer of DNA and disease via insects were to be absent or possibly controlled via processing, the need for containment would be less relevant. Other processing methods such as enzymatic hydrolysis should also be assessed [40].
  b. Species differentiation. Farmed animals are defined as one category and in a broad sense. This definition includes animals for food and non-food production (fur animals). Clear differentiation is made between ruminant and non-ruminants in terms of permitted feed materials, but this broad definition necessitates highly comparable routes for gradual relaxations for most farmed animals despite biological differences. We propose that this broad definition of farmed animals is further diversified to different groups and species of animals, primarily classified according to their feeding behaviour and their susceptibility to TSEs. We propose that a new, diversified classification of farmed animals is used, specifically: herbivores susceptible to TSEs (ruminants); minor herbivores (horses and relatives, rabbits); omnivorous and non-herbivorous terrestrial animals (pigs, poultry); vertebrate aquatic animals (fish); invertebrate aquatic animals (crustaceans, molluscs); and other invertebrates (insects, ringed worms). The latter group (invertebrates, most notable insects) could feed on a variety of different materials depending on the biological dietary preferences of the specific species in question, such as wood, manure and soil. The species-to-species ban can be applied less strictly to some extent for animals in the proposed classes with a larger genetic distance from humans (invertebrates, fish), due to a minimal risk in transmission of TSEs. Some reared insect species can exhibit cannibalism-like behaviour under certain (natural) conditions such as nutritional stress, but this has been suggested as a potential transmission route for spread of disease in such facilities [41]. Although research on that behaviour has focused on spread via cannibalism of infected live hosts rather than via (processed) protein meal of the same species, this area, and potential consequences in terms of disease proliferation is largely under-investigated. The large-scale recycling of insect proteins from the same species should therefore not be encouraged. This may need to be extended to insect species within the same taxonomic family or order. This type of cannibalism has also been observed for fish [42,43] and other aquaculture animals [44], for which similar rules should apply.
  c. Ruminant hydrolysed proteins. Hydrolyzation is a process that results in severe modification of proteins and peptides (van Raamsdonk et al., in press; [45]. After proof of sufficient safety (inactivation of zoonoses, prions), all ruminant material should be allowed to be hydrolysed and used as feed ingredient, with the exception of Specified Risk Material as defined in Article 3(1)(g) of Regulation (EC) No 999/2001. The required method development for both authenticity (rate of hydrolysation) and identification of source animal is currently being finalized.
  d. Ruminant blood products: The use of blood products derived from ruminants for non-ruminant feeds is prohibited (Regulation (EC) 999/2001, Annex IV, Chapter 2 (b)(iv)). These products (dried/frozen/liquid plasma, dried whole blood, dried/frozen/liquid red cells, haemoglobin powder) are highly processed and derived versions of animal by-products. Use as feed ingredient for non-ruminants should be considered.

**Fig. 1:**
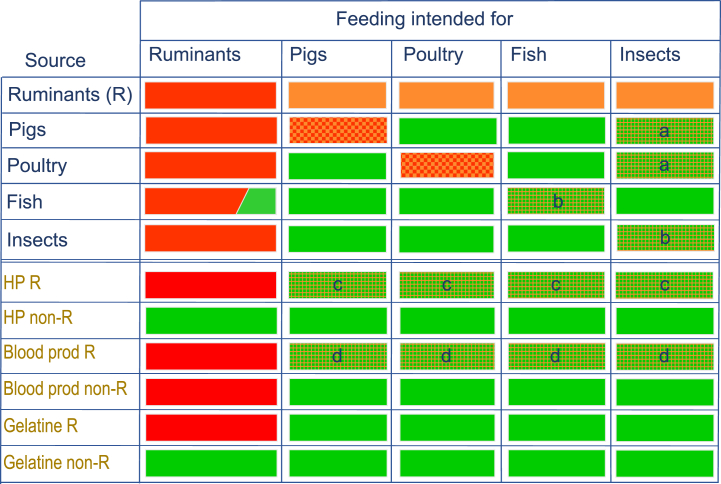
The feed bans for processed animal proteins with options for future relaxation. Index numbers refer to the text of item 1 in section 5. Red: ruminant ban; orange: extended feed ban, red dotted with orange or green: species-to-species ban; green: legalised. Orange text: extracted from EU Road map (EC, 2011). HP: hydrolysed proteins; R: ruminants. (For interpretation of the references to colour in this figure legend, the reader is referred to the Web version of this article.)
2. Increased use of alternative processing methods.
  I. Hydrolysation. Material belonging to Category 3 (a) to and inclusive of (l) can be subjected to a process of hydrolysation (Regulation (EU) 142/2011, Annex X, Chapter II, Section 5). This includes all former food products containing animal proteins still fit for human consumption – but discarded for economic reasons or packaging defects. Hydrolysation is a process that results in severe modification of proteins and peptides, generating derived products which should be subject to neither the extended feed-ban nor the species-to-species ban, since these products are excluded from the definition of PAPs (van Raamsdonk et al., in press). As discussed in section 2, at this time, certain subcategories of Category 3 – most importantly subcategory (m) [*parts of Rodentia and Lagomorphia*] – are not allowed to be used as basis for the production of gelatine, hydrolysed, or dicalcium or tricalcium phosphate. We advocate for these options being reassessed. See also item 1c above.
  II. Fermentation, as many other production procedures, is a legally recognised method, but fermented materials do not have a separate position within the animal by-products legislation aside from petfood. Since fermentation severely alters the structure of the protein, it could be recognised as a process resulting in products which would not be subjected to the species-to-species ban (part of Regulation (EU) 142/2011, Annex X). The subcategories (m), (n), and (o) of Category 3 discussed in the item above concerning hydrolysation may also be suitable for fermentation.
  III. Finally, other novel processing options might result in pure (technical) materials, such as vitamins, minerals, or specific fatty acids that could be used to supplement animal feed. A risk assessment should be conducted to determine whether the resulting specified materials should be subjected to the species-to-species ban. Research on production of new technical materials from novel food and feed source should be encouraged and funded.
3. The standard technical limit for monitoring methods (Regulation (EC) 152/2009, Annex VI: 0.1%) could be diversified depending on the type of material. Higher contamination levels could apply to single unmixed materials, intended as feed ingredient. After mixing in the final feed, the initial contamination should reach a final level below 0.1%. Initial contamination levels of up to 2% have been evaluated in a Quantitative Risk Assessment [46]. Examples are a PAP of a specified source species containing PAP material of another source species, and vegetal ingredients containing minor levels of animal proteins. The aim of a final contamination level, i.e., for compound feeds ready for the intended feeding, would comply to the species-to-species ban. This ban is based on both ethical principles (avoiding cannibalism) and prevention of zoonotic diseases. Higher limits of detection for single unmixed animal products would only be acceptable in the framework of sufficient risk management.
4. Catering waste is prohibited in a general sense (Regulation (EC) 1069/2009, article 11, part 1 (b)). Catering waste is currently defined as: “all waste food, including used cooking oil originating in restaurants, catering facilities and kitchens, including central kitchens and household kitchens” (Regulation (EU) 142/2011). This definition shows, in contrast to all other subcategories of Category 3, that catering waste is a mixture of products of plant and animal origin. A distinction should be made between professional and domestic handling of food, and between the resulting types of materials. Other countries such as Korea and Japan have shown that incorporating catering waste into animal diets can be done safely if appropriate controls are implemented [47,48].
  I. Food materials which have not been served to consumers in restaurants or canteens - i.e., those materials that have not left the kitchens of restaurants, catering facilities or other institutions – should be treated in the same manner as ‘former foodstuffs’ from industrial facilities and thus become available for processing as a feed ingredient. Proper assurance of all safety measures, the ruminant ban and the species-to-species ban remain applicable, unless processing results in products which should not be subjected to these bans (e.g., hydrolysation). Correct and sufficient handling by the food business operator of the establishment have to be demonstrated; and the operator and/or processor would also have to register with the national authority as a feed producing company; in line with current requirements for food companies producing former foodstuffs for feed.
  II. Domestic food waste and all food waste resulting from finally prepared and served dishes in restaurants and other catering facilities are currently prohibited as feed ingredient. In a full circular agronomy, these types of food products should be eligible for specified reuse, reprocessing or remanufacturing as feed ingredient. Proper assurance of all safety measures, the ruminant ban and the species-to-species ban remains applicable, unless processing results in products which are not subjected to these bans (e.g., hydrolysation). Monitoring and containment options would need to be developed.

## 6 Concluding remarks

In this article, we have presented a list of potential relaxations that provides a range of opportunities for valorisation of animal by-products of the food production chain. In the view of the major transition towards full sustainability and circularity of the feed industry, major steps in the application of animal by-products are needed. Ideally, the currently highly complex system of rules on use of animal proteins is reworked and simplified substantially, but small amendments may also allow for more circular opportunities. Promising instruments for higher valorisation of animal by-products are biological solutions, predominantly a diversification and extension of commodities for rearing insects; and technological solutions, primarily hydrolysation for producing modified peptides not reflecting the characteristics of the original proteins. Especially for the emerging EU insect rearing sector, a wider variety of suitable feed materials should be permitted so as to avoid needing to compete with feed for conventional livestock animals – thereby negating the insects’ potential for valorising products otherwise unsuitable for feed. Finally, we have argued that several current ‘zero-tolerance’ limits can be relaxed by permitting the presence of certain materials to some degree, which is anticipated to result in less waste.

## Author contribution statement

All authors listed have significantly contributed to the development and the writing of this article.

## Funding statement

This work was supported by Netherlands Ministry of Agriculture, Nature and Food Quality, Topsector AgriFood LWV19091 (BO-64-001-013).

## Data availability statement

No data was used for the research described in the article.

## Declaration of interest’s statement

The authors declare no conflict of interest.
